# Synthesis, Characterization and Nanoformulation of Novel Sulfonamide-1,2,3-triazole Molecular Conjugates as Potent Antiparasitic Agents

**DOI:** 10.3390/ijms23084241

**Published:** 2022-04-11

**Authors:** Faizah S. Aljohani, Nadjet Rezki, Mohamed R. Aouad, Bassma H. Elwakil, Mohamed Hagar, Eman Sheta, Nermine Mogahed Fawzy Hussein Mogahed, Sanaa K. Bardaweel, Nancy Abd-elkader Hagras

**Affiliations:** 1 Department of Chemistry, College of Science, Taibah University, Al-Madinah Al-Munawarah 30002, Saudi Arabia; nadjetrezki@yahoo.fr (N.R.); aouadmohamedreda@yahoo.fr (M.R.A.); 2 Department of Medical Laboratory Technology, Faculty of Applied Health Sciences Technology, Pharos University in Alexandria, Alexandria 21321, Egypt; bassma.hassan@pua.edu.eg; 3 Department of Chemistry, Faculty of Science, Alexandria University, Alexandria 21321, Egypt; mohamedhaggar@gmail.com; 4 Department of Pathology, Faculty of Medicine, Alexandria University, Alexandria 21321, Egypt; emansheta@yahoo.com; 5 Department of Parasitology, Faculty of Medicine, Alexandria University, Alexandria 21321, Egypt; nermine_mogahed@yahoo.com; 6 Department of Pharmaceutical Sciences, School of Pharmacy, University of Jordan, Amman 11942, Jordan; s.bardaweel@ju.edu.jo

**Keywords:** 1,2,3-triazoles, sulfadiazine analogues, nanoformulation, *Toxoplasma gondii*

## Abstract

*Toxoplasma gondii* (*T. gondii*) is a highly prevalent parasite that has no gold standard treatment due to the poor action or the numerous side effects. Focused sulfonamide-1,2,3-triazole hybrids **3a–c** were wisely designed and synthesized via copper catalyzed 1,3-dipolar cycloaddition approach between prop-2-yn-1-alcohol **1** and sulfa drug azides **2a–c**. The newly synthesized click products were fully characterized using different spectroscopic experiments and were loaded onto chitosan nanoparticles to form novel nanoformulations for further anti-Toxoplasma investigation. The current study proved the anti-Toxoplasma effectiveness of all examined compounds in experimentally infected mice. Relative to sulfadiazine, the synthesized sulfonamide-1,2,3-triazole (**3c**) nanoformulae demonstrated the most promising result for toxoplasmosis treatment as it resulted in 100% survival, 100% parasite reduction along with the remarkable histopathological improvement in all the studied organs.

## 1. Introduction

*Toxoplasma gondii* (*T. gondii*) is a globally spread foodborne parasite that causes a disease known as toxoplasmosis, infecting about 50% of the world’s population [1,2]. There are three strains of *T. gondii* distributed according to their virulence. Type I strain (e.g., RH strain) owes the highest virulence and is lethal at all doses, in all strains of mice, during the acute proliferative stage of the disease [3,4,5]. The parasite has three main life cycle stages; the rapidly multiplying tachyzoite (Greek tachos = speed), the encysted transmissible stage found in tissues known as bradyzoite (Greek brady = slow) and the sexual stage occurring in the feline intestine called oocyst [6,7]. *T. gondii* may infect the definitive host by more than one pathway; ingestion of undercooked meat of an animal infected with tissue cysts, ingestion of unclean vegetables contaminated with oocyst or it can be transmitted during pregnancy from an infected mother to her fetus [2,8,9]. *Toxoplasma* infection can be classified into acute or chronic stage according to the speed of infection. The acute stage occurs as a result of the rapidly proliferative tachyzoites where the cell penetration requires the attachment between the conoid (anterior tip of tachyzoite) and the host cell [2,3]. Tachyzoites can nearly invade all body tissues such as the liver, spleen, brain, muscles, lung, eye and placenta [2]. Most infections are asymptomatic, although they might be accompanied by fever, lymphadenopathy, muscular discomfort and headache [3]. On the other hand, the disease is strongly associated with lethality in immunocompromised or congenitally infected patients [2,3,8,9].

Despite the diversity of the currently available drugs for Toxoplasma, unfortunately, most of them have poor effectiveness and are associated with many side effects, in addition to their poor penetration to biological barriers such as the blood-brain barrier [2,10,11]. The first line of therapy used to treat toxoplasmosis relies mainly on the inhibition of folate synthesis pathway. Sulfadiazine is one of the sulfonamide groups acting on the folate synthesis pathway which is essential for the parasite to form pyrimidine bases (cytosine, thymine) in its DNA [12]. Sulfadiazine has relatively high tissue permeability; however, the low water solubility may negatively affect its absorption [13,14]. Nitrogen-containing heterocyclic compounds, particularly 1,2,3-triazoles [15], have aroused the interest of medicinal chemists in the creation of new therapeutic candidates due to its fascinating biomedical applications and numerous pharmacological properties such as antibacterial [16], antiviral [17], antifungal [18], antimalarial [19], anti-HIV [20], antiallergic [21], antitubercular [22], CNS depressant [23], analgesic [24], anticonvulsant [25], antihypertensive [26] and antiproliferative agents [27].

The molecular hybridization method, in which two or more distinct active pharmacophores are linked together with or without the use of a linker, is a simple way to discover new drugs [28,29,30]. This method is currently the most frequently used in the development of new pharmacological scaffolds that target multiple sites [30]. As a result, hybrid molecules can reduce the possibility of drug-drug interactions as well as multiple drug resistance. Due to the extreme biological significance of the tunable 1,2,3-triazole core and sulfonamide moieties, we disclose herein the development of some sulfonamide connected 1,2,3-triazole hybrids as a continuation of our interest in the development of such hybrid molecules and the investigation of their synergetic effect [31,32,33,34,35,36,37,38,39,40,41,42,43,44].

Nanotechnology gained greater attentions after proving its efficacy in improving the drugs’ pharmacokinetic profile. This includes enhancing the solubility, the dissolution rate, the stability and above all modulating the drug permeability through absorption into membranes leading to reduced drug doses [45]. One of the main goals of using nanoformulations is related to their ability to enhance the penetration of the therapeutic agents through biological barriers such as the blood–brain barrier (BBB) [46]. During the past decades, chitosan has gained great attention and has been broadly applied in pharmaceuticals development with respect to its excellent biological characteristics of biocompatibility, absorptivity, non-hypersensitivity, biodegradability and wound healing properties [47]. Numerous studies proved the effectiveness of chitosan nanoparticles as anti-parasitic agent. Said et al. [48] reported the anti-*Giardia lamblia* effect of chitosan nanoparticles. Another study showed the promising anti-*Toxoplasma* effect of chitosan nanoparticles [49].

In the present study, the therapeutic efficacy of sulfonamide 1,2,3-triazole hybrids and their novel nanoformulations were assessed in comparison to the gold standard sulfadiazine in a murine model of acute virulent toxoplasmosis.

### Rationale Study

Toxoplasmosis is deemed as one of the most serious diseases currently affecting humans. Over the last decade, various investigations for disease prevention have been envisioned against the tachyzoite form of *T. gondii*.

**Figure 1:**
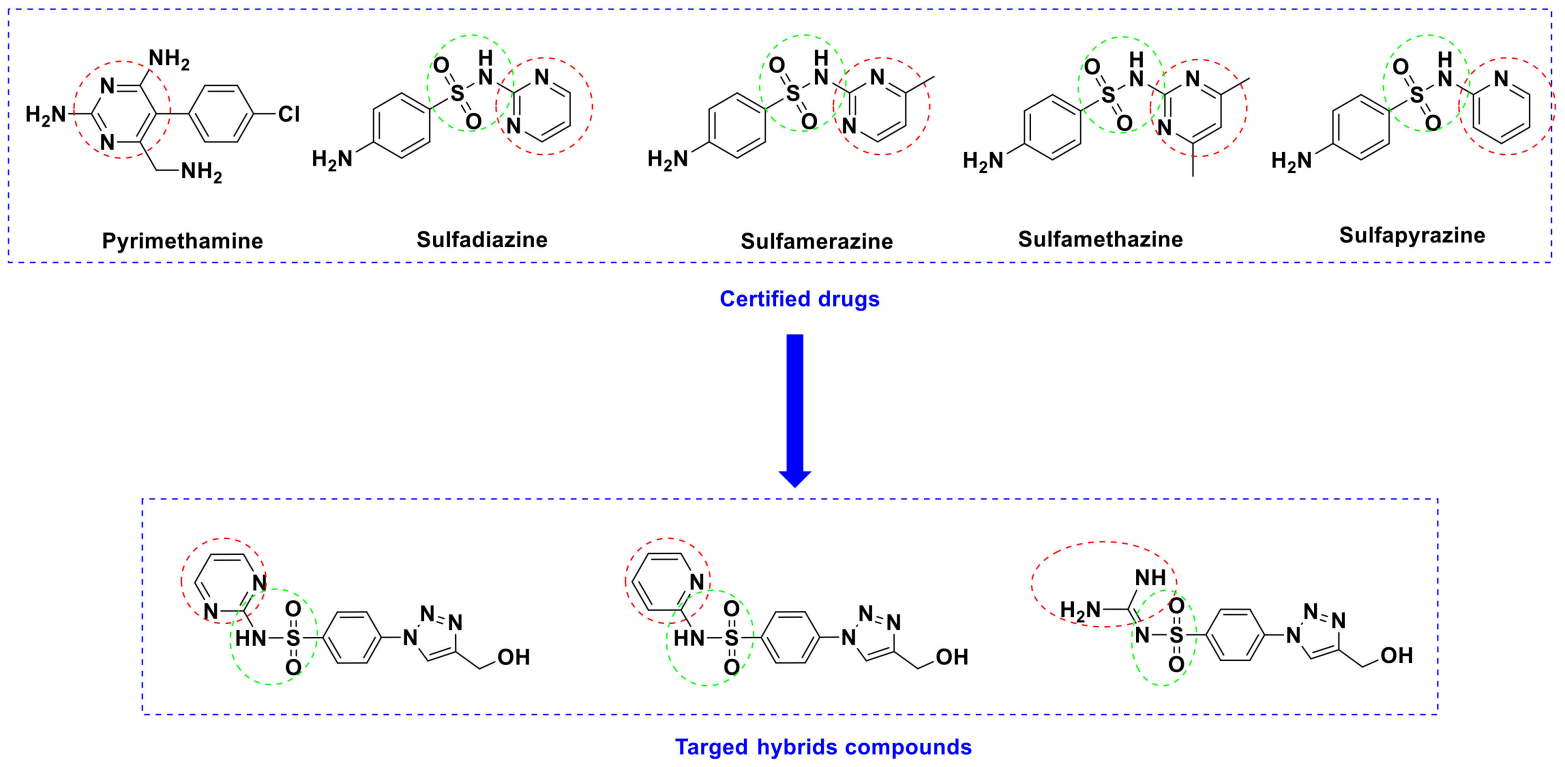
Schematic representation of the rational design.

The modeling analysis revealed that pyrimethamine, which is found in the vast bulk of medication regimens, is the most effective scaffold. However, the most effective treatment molecular hybrids currently available are derived from pyrimethazine such as sulfadiazine, sulfamerazine, sulfamethazine and sulfapyrazine (Figure 1). These attractive scaffolds prompted us to design and synthesize focused 1,2,3-triazole-sulfonamide molecular conjugates as mimic of corresponding features in the proposed scaffold, which will be used for further investigation and evaluation of potential inhibition against *T. gondii.*

## 2. Results and Discussion

### 2.1. Chemistry

#### Synthesis and Characterization

**Scheme 1:**
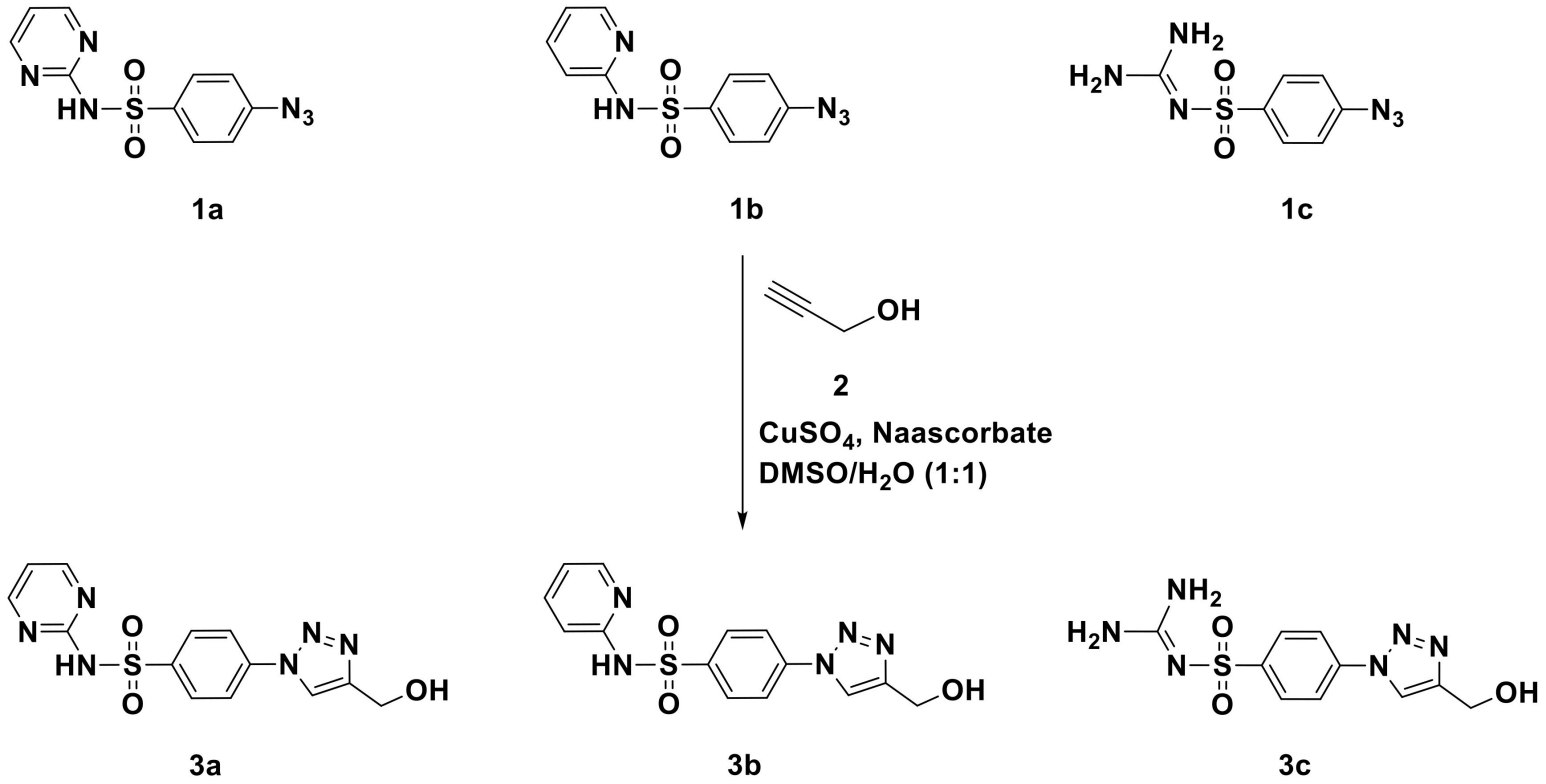
Synthesis of 1,2,3-triazole-sulfonamide molecular conjugates **3a–c**.

The strategy adopted for the synthesis of the focused sulfoamide-1,2,3-triazole hybrids **3a–c** was depicted in Scheme 1. The most straightforward process for preparing 1,2,3-triazoles-sulfoamide molecular hybrids is by using a direct and an efficient Cu(I)-catalyzed azide-alkyne cycloaddition strategy (CuAAC), as documented in the literature [50]. By adopting this approach, new 1,2,3-triazole-sulfoamide molecular conjugates **3a–c** were designed and synthesized through a copper-mediated 1,3-dipolar cycloaddition between prop-2-yn-1-alcohol **2** as an alkyne and appropriate sulfa drug azides **1a–c** as an organic azide (Scheme 1), in the presence of copper sulfate and sodium ascorbate as catalysts. It was noticeable that the used sulfa azides were obtained through the diazotization of their selected sulfa drugs followed by their treatment with sodium azides according to the reported literature [50].

**Figure 2:**
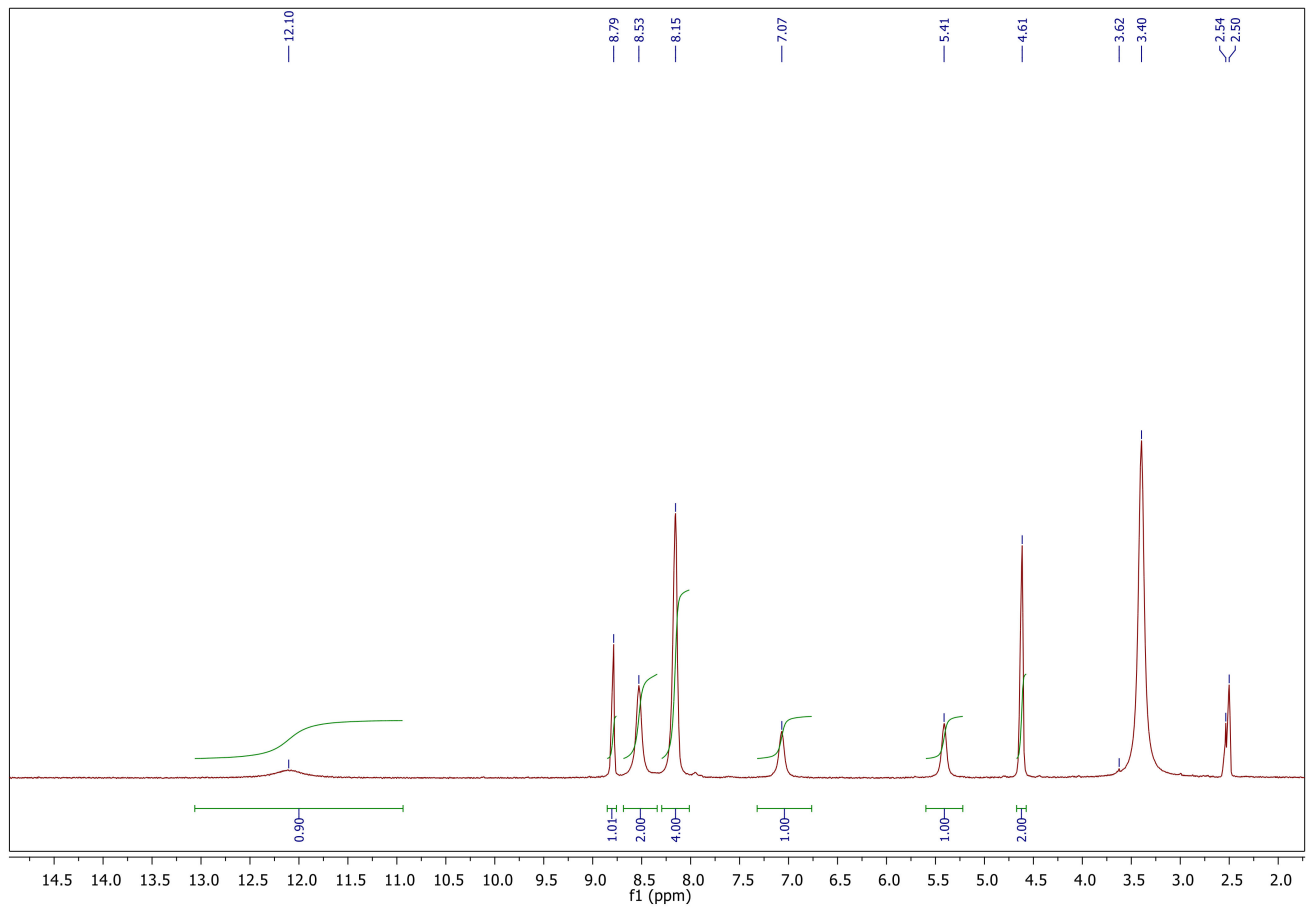
^1^H NMR Spectrum of the 1,2,3-triazole-sulfoamide molecular conjugates **3a**.

The structures of the resulting 1,2,3-triazole-sulfonamide molecular conjugates **3a–c** were elucidated based on their spectral data. The spectral data of the click adduct **3a** was taken as the model to discuss the success of the click reaction. Thus, its IR clearly showed the absence of the absorption bands of the alkyne groups (C≡C–H) and azides (N_3_) of its corresponding starting materials **1** and **2a**, and the presence of new absorption bands at 3487 cm^−1^ of the lateral hydroxyl group. On the other hand, the investigation of its ^1^HNMR data (Figure 2) confirmed the success of the click synthesis and exhibited the disappearance of the acetylenic protons (≡C–**H**) of the propargyl side chain and appearance of the distinct singlet at δ_H_ 8.79 ppm attributed to the H-5-triazolyl supporting the formation of triazole ring of **3a**. New signals were recorded at δ_H_ 8.53–7.07 ppm belonging to the aromatic protons, 5.41 ppm assigning O**H** protons and δ_H_ 4.61 ppm attributed to the methylene protons (C**H_2_**).

**Figure 3:**
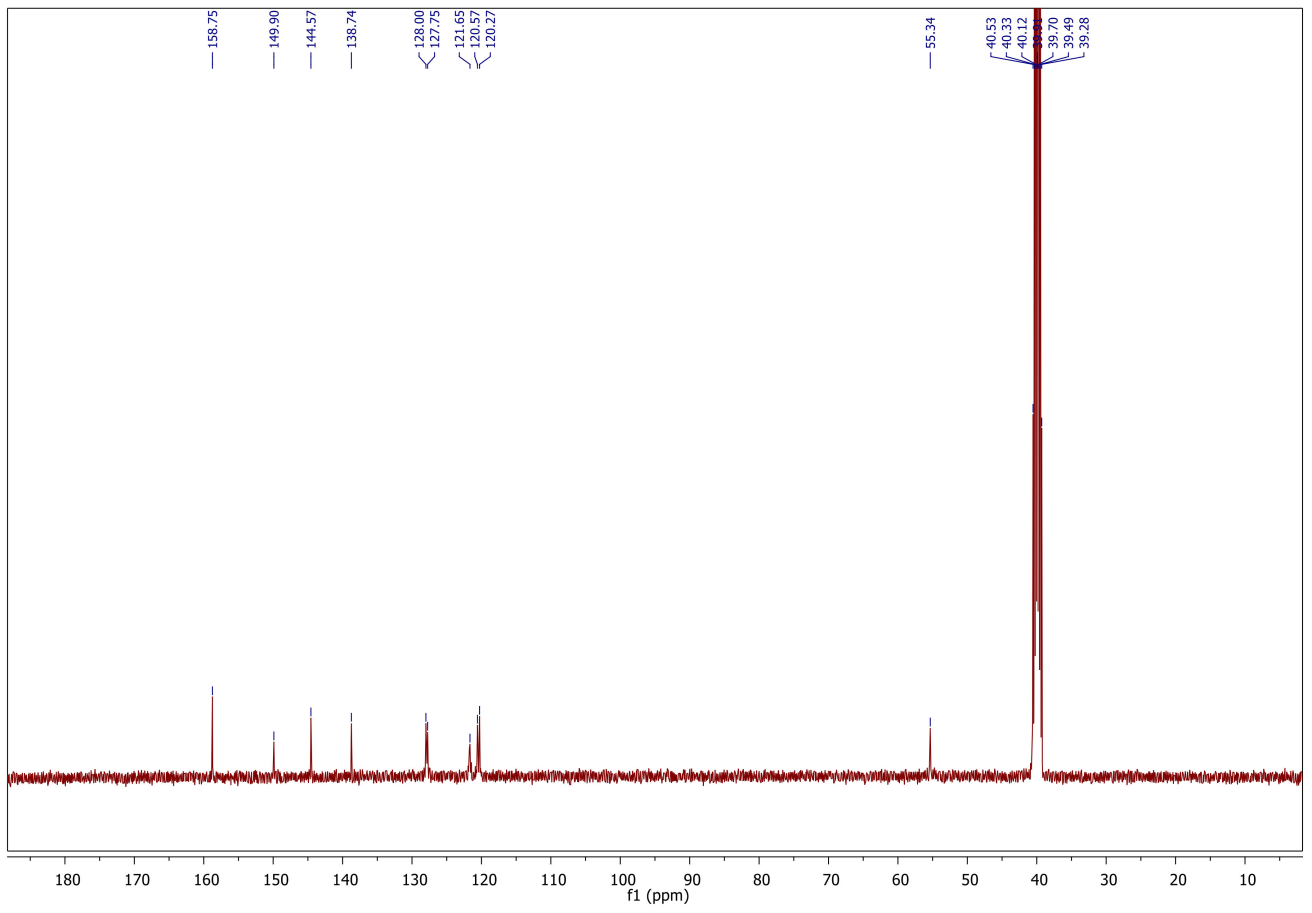
^13^C NMR Spectrum of the 1,2,3-triazole-sulfoamide molecular conjugates **3a**.

Moreover, the ^13^C NMR analysis (Figure 3) revealed the absence of the Sp-carbons of the starting alkyne and presence of new aromatic and imine carbons between δ_C_ 158.95–120.27 ppm (See experimental section).

### 2.2. Formulation

#### Nanoparticles Characterization

**Figure 4:**
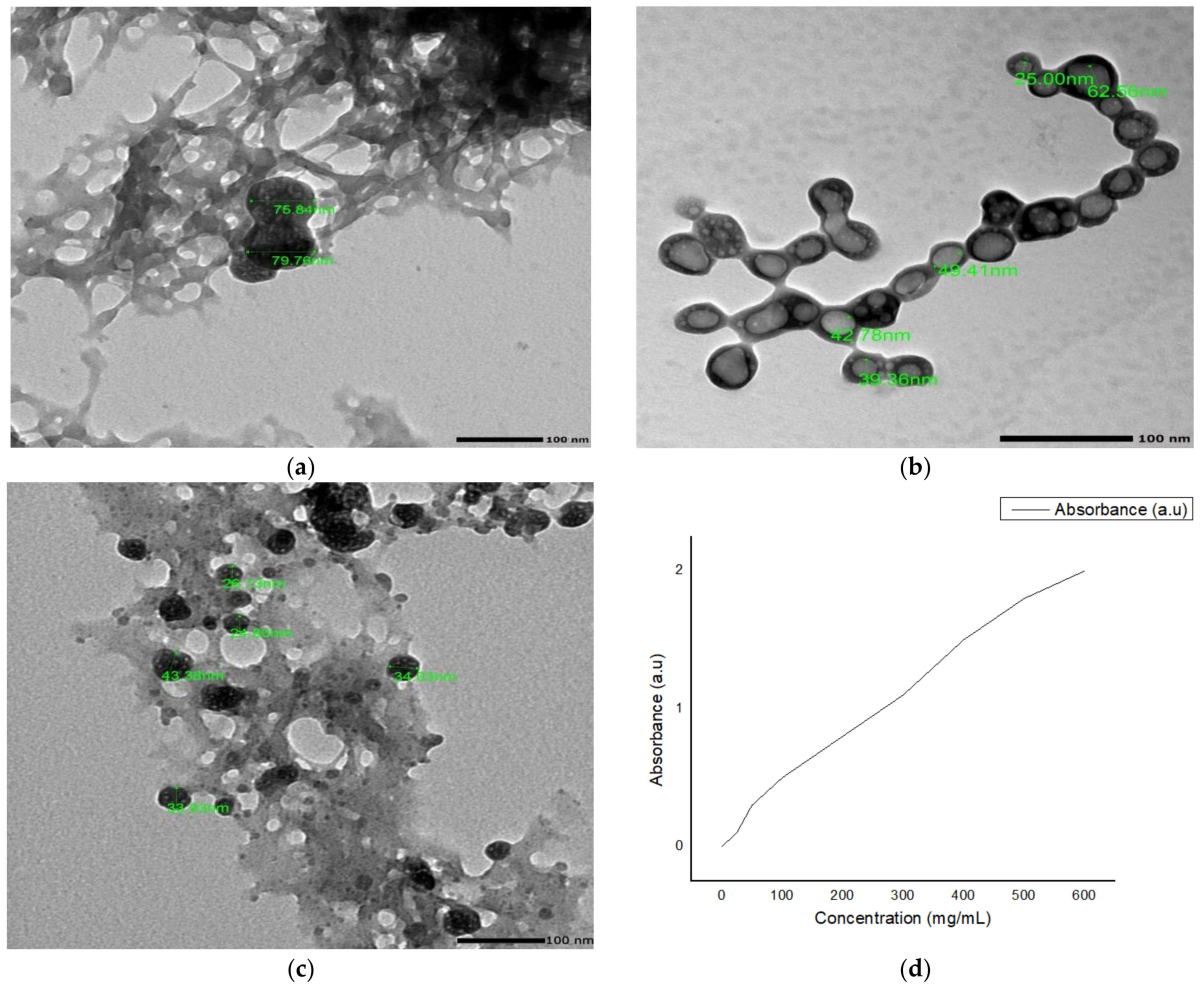
TEM micrographs of **NCs-3a** (**a**), **NCs-3b** (**b**), **NCs-3c** (**c**) nanoparticles (100,000×) and (**d**) calibration curve of the synthesized sulfonamide-1,2,3-triazole molecular conjugates.

The newly synthesized chitosan loaded 1,2,3-triazole-sulfoamide nanoparticles were prepared through ionic gelation method. Data in Table 1 revealed that the loaded nanoparticles had a positive charge reflecting the stability of the prepared formulae that may be attributed to the nature of the loaded compounds. The prepared nanoparticles had a regular, smooth and spherical shape with average sizes of 76.3, 50.4 and 36.0 nm for NCs-3a, NCs-3b and NCs-3c nanoparticles, respectively, as illustrated in Figure 4. The highest entrapment and loading efficiencies were noticed with NCs-3c nanoparticles (89.27 and 43.31%, respectively) while NCs-3a and NCs-3b showed inferior results (Table 1).

### 2.3. Biological Assessment of the Novel Sulfonamide Derivatives

#### 2.3.1. Clinical Behavior of Infected Mice

Infected untreated mice revealed a marked reduction in food intake with lethargic attitude, hunched posture and ruffled fur on the fifth day post-infection. On contrary, infected treated mice seemed comparatively healthy with good food intake. The uppermost activity of mice was observed among subgroup **IIf**, receiving nanoparticles loaded with the triazole-sulfonamidederivative **3c**. These observations are in worthy agreement with the previous studies [2,51].

#### 2.3.2. Parasitological Study

##### Survival Time

In the present work, three crude triazole-sulfonamidederivatives **3a–c** and their nanoformulae were evaluated in addition to the commercially available sulfadiazine for the treatment of mice infected with *T. gondii* virulent RH strain. Survival time was observed for 30 days post-infection for all subgroups where a high significant difference in mice survival was observed between treated and untreated subgroups (*p* < 0.001). The death of infected untreated mice (subgroup Ia) started on the sixth day post-infection with no survivors beyond the seventh day post-infection representing a mean survival time of 6.6 days. Likewise, several studies reported the same finding. The rapid death of infected mice may be clarified by the extensive spreading of the virulent *Toxoplasma* strain [2,51,52]. Difference in survival time of mice between the current work and other studies may be attributed to different infection doses, type of strain used, route of infection, type of drug used and mode of administration along with dose and duration of management [2,49,51].

**Figure 5:**
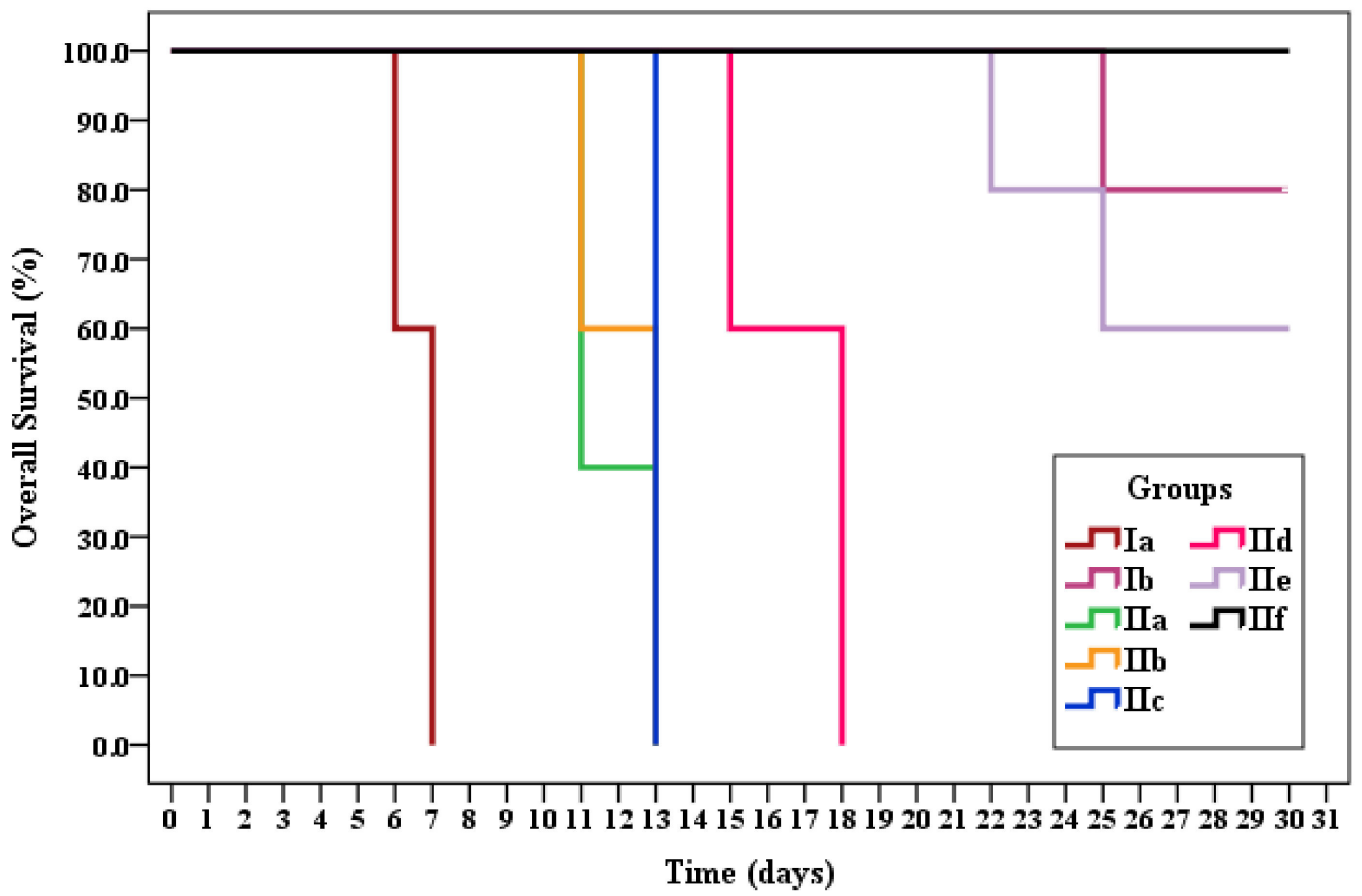
Kaplan-Meier survival curve for all subgroups.

All the treated mice revealed a significant increase in the mean survival time in comparison to the untreated control group; they indicated a mean ranging from 11.8 to 30 days. Subgroup IIf (mice treated with nanoparticles loaded with the triazole-sulfonamidederivative **3c**) showed the longest survival time, whereas 100% of mice survived till the end of the study exceeding the control subgroup Ib treated with sulfadiazine (Table 2 and Table 3 and Figure 5).

##### Parasite Load and Percent Reduction (%R)

**Figure 6:**
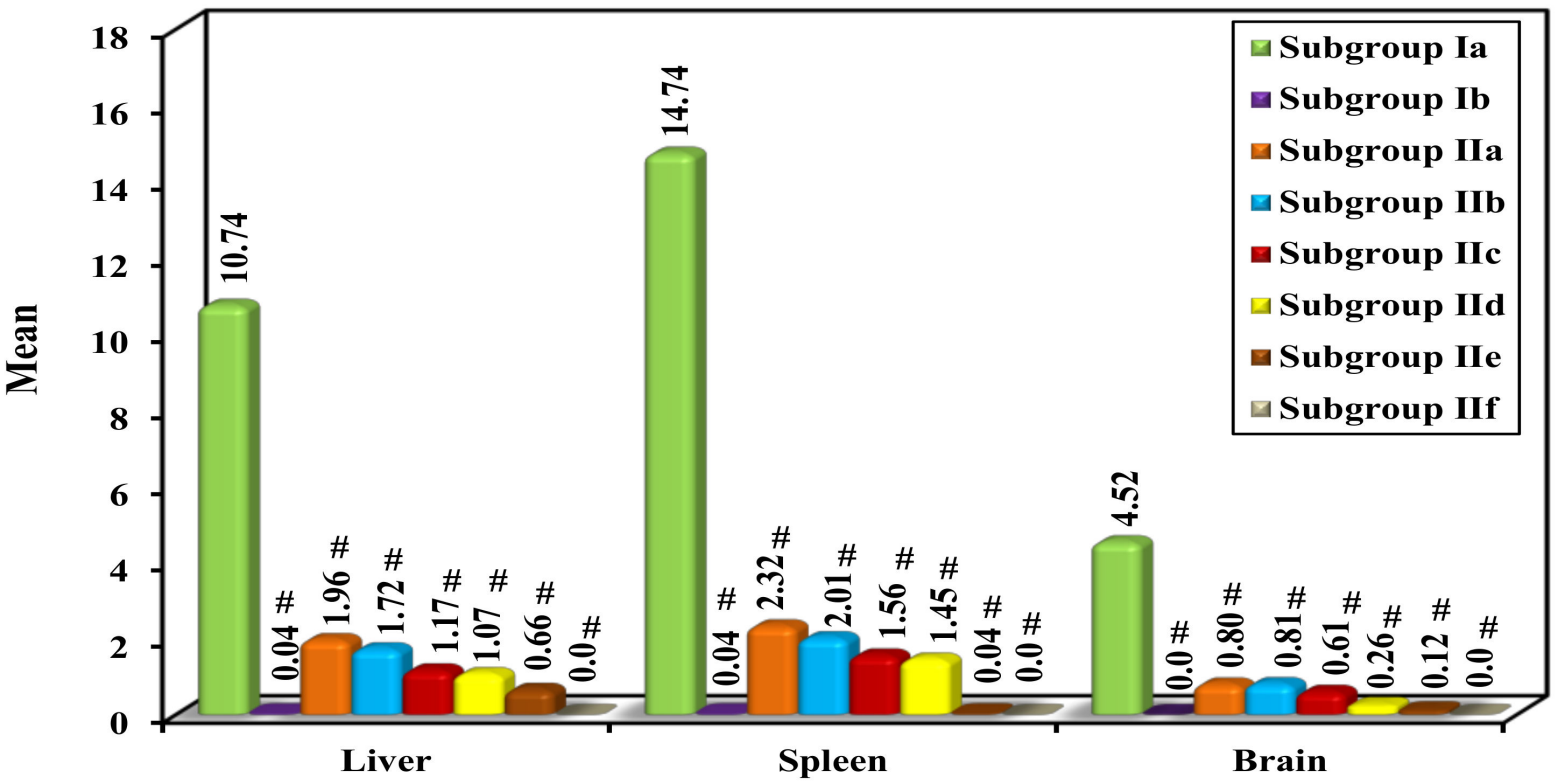
Mean tachyzoites count in the liver, spleen and brain of the different studied subgroups. #: Significant with subgroup **Ia**.

**Figure 7:**
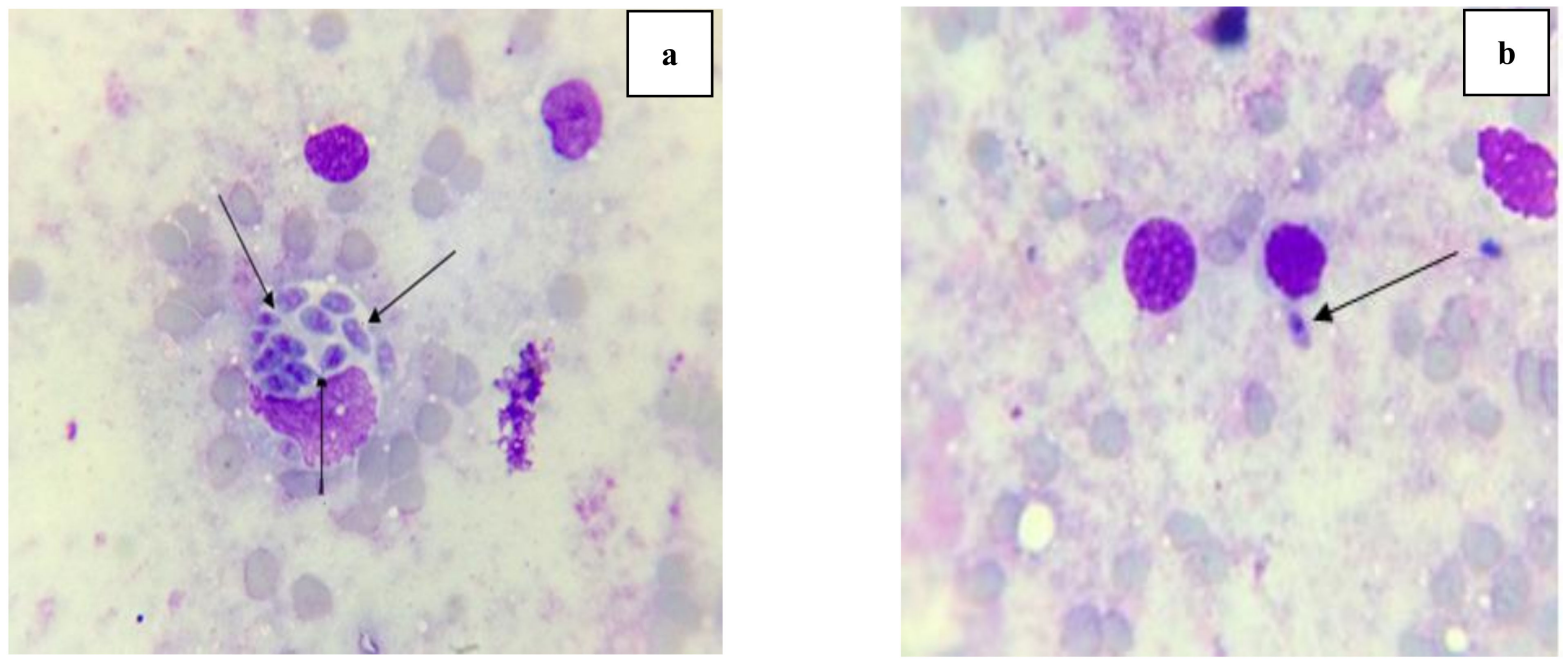

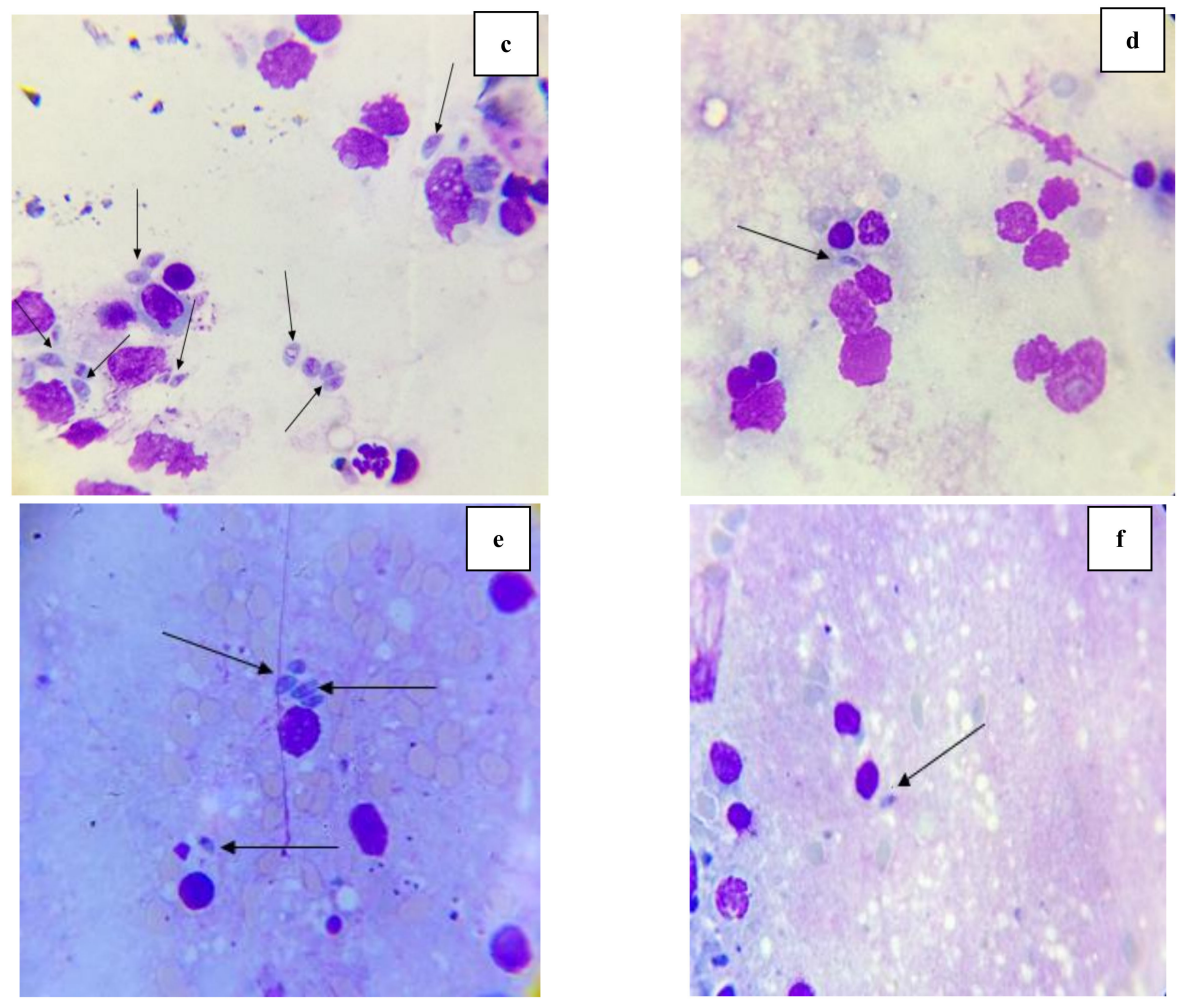
Giemsa-stained impression smears of liver, spleen and brain showing *Toxoplasma* tachyzoites in infected untreated control and infected treated mice. (**a**): *T. gondii* tachyzoites in infected untreated control liver (Giemsa stain ×1000). (**b**): *T. gondii* tachyzoites in infected treated liver (Giemsa stain ×1000). (**c**): *T. gondii* tachyzoites in infected untreated control spleen (Giemsa stain ×1000). (**d**): *T. gondii* tachyzoites in infected treated spleen (Giemsa stain ×1000). (**e**): *T. gondii* tachyzoites in infected untreated control brain (Giemsa stain ×1000). (**f**): *T. gondii* tachyzoites in infected treated brain (Giemsa stain ×1000).

A high statistically significant difference between all groups regarding parasite load in the different studied organs was observed (AVOVA test, *p* < 0.001). The mean tachyzoites count in the liver, spleen and brain of the infected untreated control was 10.74, 14.74 and 4.52/20 oil immersion field (OIF), respectively. Post hoc pairwise comparison showed significant reduction in the parasite load between the infected untreated control and each of the other treated subgroups. Similar observation was reported by Alves and Vitor [53] who indicated the effectiveness of sulfadiazine in the treatment of toxoplasmosis [53]. Concerning the treated mice, the lowest mean tachyzoites count (highest percent reduction) was observed among subgroup **IIf,** which successfully reached 100% reduction in parasite load achieving better results than the control subgroup **Ib** treated with sulfadiazine (Table 4 and Figure 6 and Figure 7). The observed reduction indicates successful tissue penetration and blood–brain barrier passage of the used formula. This coincides with several studies performed on drug-loaded NPs that ensured the tachyzoites reduction in different organs compared to the unloaded drug [2,52,54]. The marked difference in the biological activity between the sulfonamide analogues can be explained by the difference in the chemical composition where **3a**, **3b** and **3c** had azine, diazine and guanidine linked to substituted 1,2,3-triazole ring, respectively.

#### 2.3.3. Morphological Study of Tachyzoites by Light Microscope and Scanning Electron Microscope (SEM)

Peritoneal exudate of infected untreated mice was examined by light microscope showing normal movement of tachyzoites with no signs of distortion. The tachyzoites of subgroups **IIa**, **IIb** and **IIc** revealed slow movement, while sulfadiazine and NP-treated tachyzoites showed complete loss of movement.

**Figure 8:**
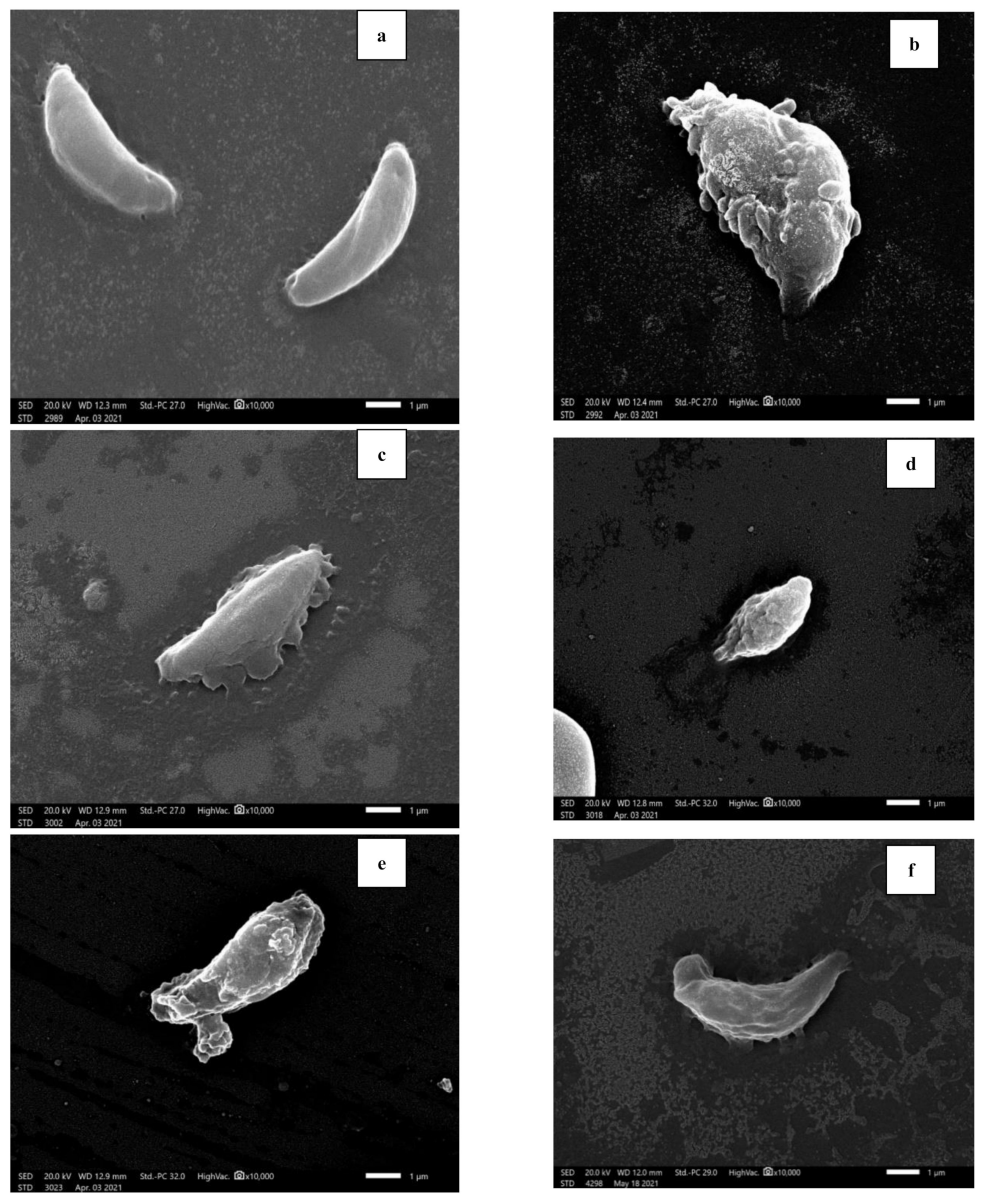

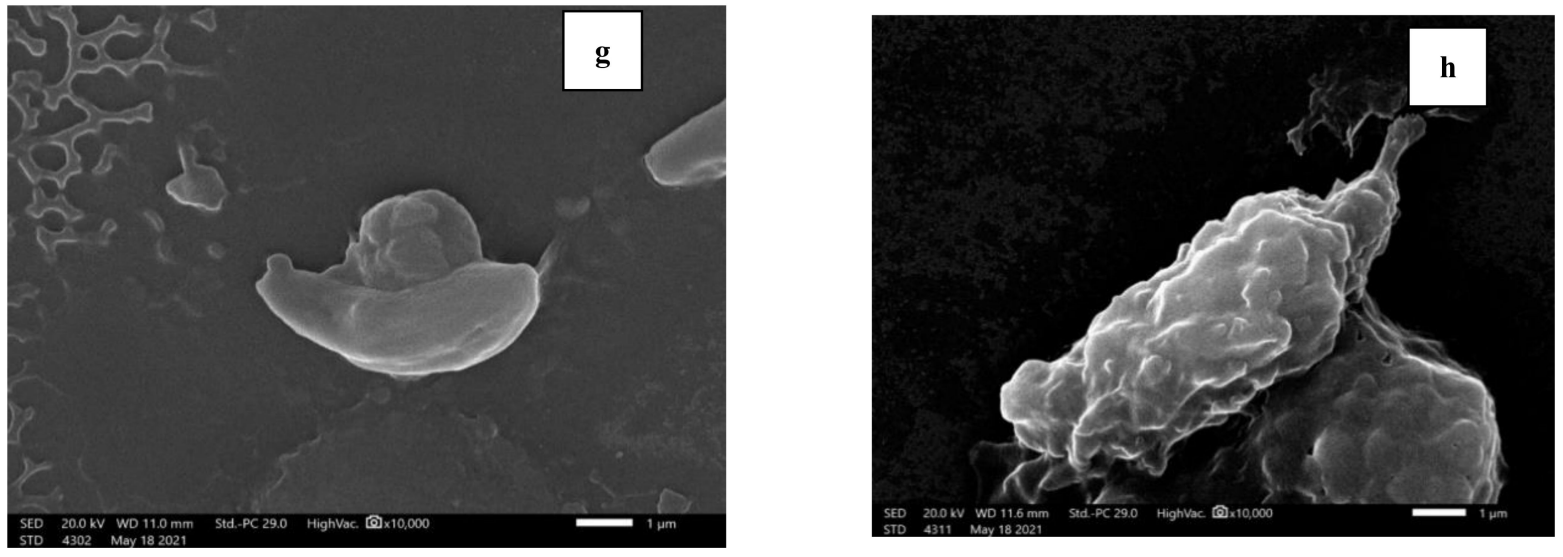
SEM of *T. gondii* tachyzoites collected from the peritoneal cavity of infected mice from different studied subgroups. (**a**): Tachyzoite from subgroup **Ia** (infected untreated control), presenting crescent shape with obvious conoid (×10,000). (**b**): Tachyzoite from subgroup **Ib**, showing evident ballooning, distortion of the crescent shape with multiple large papules on the surface and loss of the tapered ends. (×10,000). (**c**): Tachyzoite from subgroup **IIa**, showing loss in the crescent shape with disorganized lacerated outer surface (×10,000). (**d**): Tachyzoite from subgroup **IIb**, showing a shrunken distorted organism losing its crescent shape, tapered ends and conoid with marked surface erosion and ulceration (×10,000). (**e**): Tachyzoite from subgroup **IIc**, showing mutilated rapturing organism losing its conoid with disorganized lacerated surface (×10,000). (**f**): Tachyzoite from subgroup **IId**, indicating lacerated parasite losing its smooth surface (×10,000). (**g**): Tachyzoite from subgroup **IIe**, showing rapturing mutilated organism losing its conoid and smooth surface (×10,000). (**h**): Tachyzoite from subgroup **IIf**, showing disfigured rapturing parasite with marked ballooning along with damaged crescent shape and loss of the tapered ends (×10,000).

Examination of the peritoneal fluid of infected untreated mice using SEM revealed normal tachyzoites that were elongate with entirely smooth surface. They had crescent-shape possessing a rounded pole at one end and a pointed pole at the other with an obvious conoid used in parasite penetration. Nevertheless, tachyzoites collected from all treated subgroups presented loss in crescent shape, distortion of smooth surface, loss of tapered ends and/or loss of the conoid. They also showed papules, lacerations, erosions and/or ulcerations. The tachyzoite ultrastructural alterations were more apparent in subgroup **IIf** where it appeared mutilated, ballooned and almost rupturing (Figure 8). These observed changes were reported in other studies where the disorganized conoid was explained as an apparent sign of the anti-*Toxoplasma* treatment efficiency. The positively charged chitosan was reported to have the ability to form an electrostatic interaction with the negatively charged membrane of tachyzoites resulting in their malformation along with the used treatment [2,49,55].

#### 2.3.4. Histopathological Study

**Figure 9:**
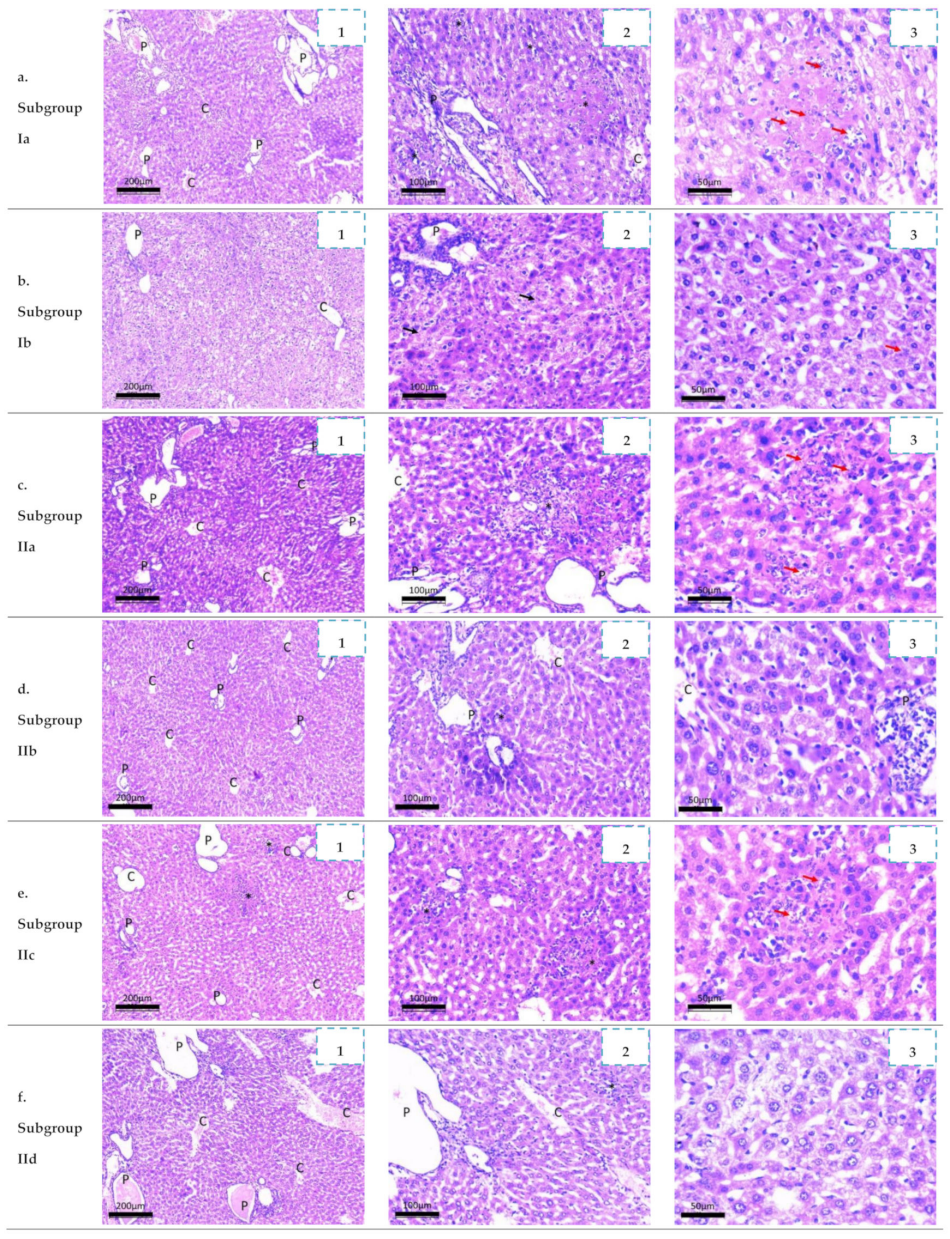

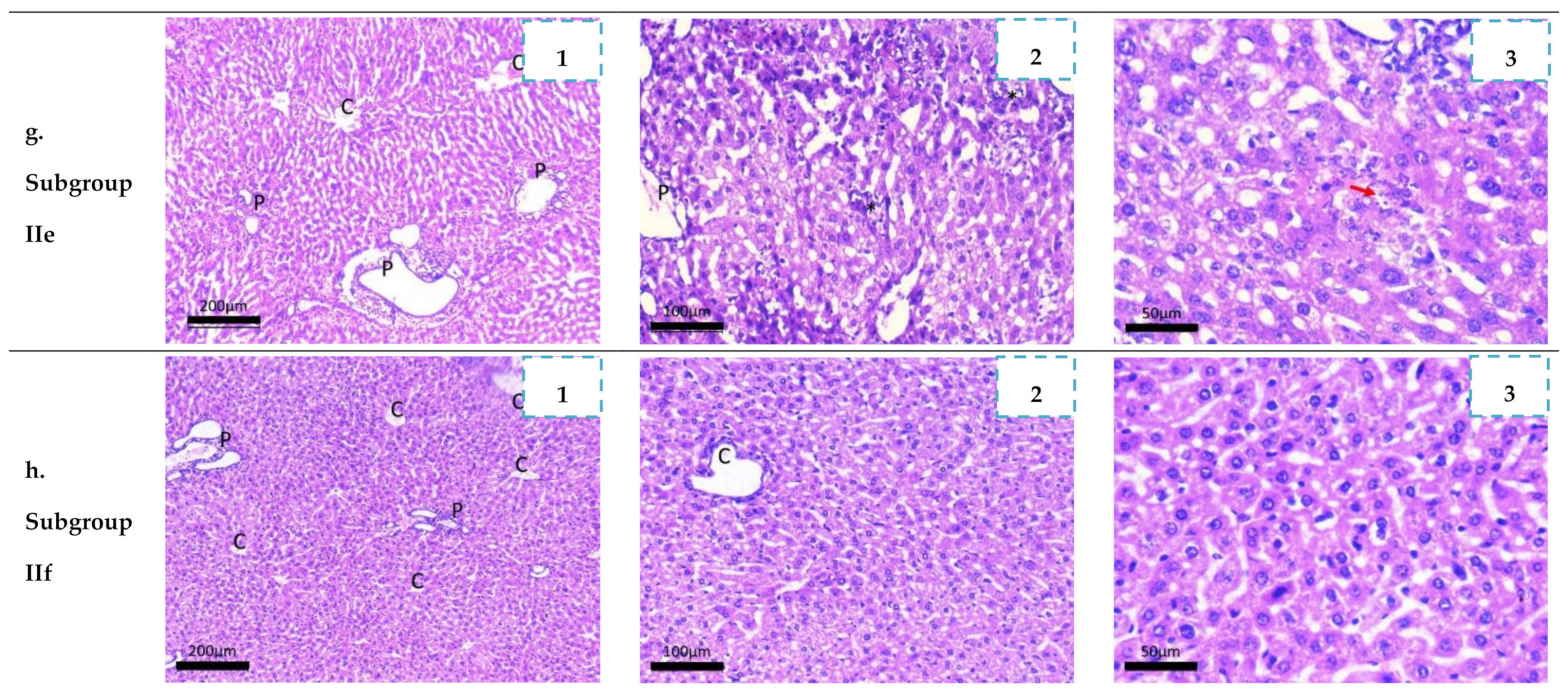
Liver sections of different *Toxoplasma*-infected subgroups showed: (**a**) Subgroup Ia: (**a1**) loss of normal lobular architecture of liver (100×). (**a2**) Moderate mononuclear portal inflammation is seen as well as dilated ectactic portal vessels (P). Multiple foci of focal lytic necrosis are seen (asterisk). They are seen as focal collection of lobular lymphocytes around apoptotic hepatocytes (200×). (**a3**) Numerous tachyzoites (red arrows) intra- and extracellular within necrotic foci (400×). (**b**) Subgroup Ib: (**b1**) Restoration of liver architecture (×100). (**b2**) Moderate portal inflammation is still detected (P) and wide areas of vacuolar degenerative changes and microvesicular steatosis (back arrows) (200×). (**b3**) Histologically free hepatocytes with occasional tachyzoites (red arrows). (**c**) Subgroup IIa: (**c1**) Loss of normal lobular architecture (×100). (**c2**) Portal tracts (P) show mild mononuclear inflammatory infiltrate and ectactic vascular spaces (200×). (**c3**) Necrotic hepatocytes with evident residual tachyzoites (red arrows). (**d**) Subgroup IIb: (**d1**) Restoration of normal architecture of liver (×100). (**d2**) Mild portal inflammation is seen (P) with mild residual vascular congestion/ectasia. Only one focus of focal lytic necrosis NPs detected (*) (200×). (**d3**) Normal histology with no tachyzoites detected in H&E staining (400×). (**e**) Subgroup IIc: (**e1**) Restoration of liver architecture (×100). Mild portal inflammation is still detected (P). (**e2**) Few foci of focal lytic necrosis are seen (*) (200×). (**e3**) Necrotic hepatocytes with evident residual few tachyzoites (red arrows) (400×). (**f**) Subgroup IId: (**f1**) Low power view showing restoration of liver architecture (×100). (**f2**) Mild portal inflammation is still detected (P) with improvement of vascular ectasia/congestion and occasional foci of focal lytic necrosis is seen (asterisk) (200×). (**f3**) Histologically free hepatocytes. No tachyzoites were detected (400×). (**g**) Subgroup IIe: (**g1**) Restoration of liver architecture (×100). No portal inflammation (P) is still detected while mild vascular congestion is still seen. (**g2**) Few foci of focal lytic necrosis are seen (asterisk) (200×). (**g3**) Necrotic hepatocytes with few rare residual tachyzoites (red arrows) (400×). (**h**) Subgroup IIf: (**h1**) Restoration of liver architecture (×100). No portal inflammation is still detected (P) or vascular congestion/ectasia. (**h2**) No focal lytic foci are seen (200×). (**h3**) Histologically normal hepatocytes with no detected tachyzoites (400×).

**a. Liver:** Microscopic examination of **subgroup Ia** liver sections showed disturbed lobular architecture of the liver. Hepatic capsule was thickened and covered by tachyzoites rich inflammatory exudate. The portal tracts showed moderate mononuclear inflammatory infiltrate with evident interface hepatitis. The infiltrate was composed mainly of lymphocytes, plasma cells, histiocytes and rare eosinophiles. Evident congestion and dilatation (ectasia) of central veins, portal vessels and sinusoids were frequently detected. Lobules showed foci of lytic necrosis [>20/×10 high power field (HPF)], diffuse vacuolar degeneration and focal microvesicular steatosis. Centrilobular necrosis was also noted. Hepatocytes showed intra- and extracellular high tachyzoites load (Figure 9a1–a3).

Liver sections of **subgroup Ib** (infected and treated with sulfadiazine) revealed evident reduction in portal inflammatory infiltrate with mild focal interface hepatitis. Vascular congestion of portal vessels and central veins were still detected. Regarding hepatocytes, focal lytic necrosis dropped to be 5/10 HPF while no centrilobular necrosis was seen. On the other hand, grade III diffuse microvesicular steatosis was detected. Rarely, *Toxoplasma* tachyzoites were seen (Figure 9b1–b3)). With regard to **subgroup IIa** (infected and treated with 3a sulfadiazine analogue) minimal improvement of hepatic histology was observed when compared to the control as focal lytic necrosis was still detected and counted up to 18/10 HPF. Portal inflammation, vascular inflammation and centrilobular necrosis were also detected (Figure 9c1–c3)). Loading those drugs on chitosan nanoparticles (subgroup IId) improved its effect in toxoplasmosis treatment. The capsular inflammation was minimal as well as portal inflammation with no interface hepatitis. Focal lytic necrosis foci were 7/10 HPF. No centrilobular necrosis or tachyzoites were detected (Figure 9d1–d3)). Mice of subgroup IIb and IIc showed marked improvement of hepatic histology. Hepatocytes were of normal morphology. They were arranged in cords and trabeculae showing eosinophilic granular cytoplasm. No steatosis or necrosis were seen. Focal lytic necrosis dropped to 12/10 HPF and 5/10 HPF, respectively. No tachyzoites were detected by light microscopy. Portal tracts showed minimal inflammation. Vascular congestion and dilatation were mild and seen in a few tracts (Figure 9e1–e3) and Figure 9f1–f3)). Loading those drugs on chitosan nanoparticles (subgroups IIe and IIf) increased their effect, as focal lytic necrosis was only 4 and 2/10 HPF, respectively. Postal inflammation was decreased especially in subgroup IIf (Figure 9g1–g3) and Figure 9h1–h3)). A similar finding was reported by Allam et al., in 2021 when spiramycin-CS NPs showed nearly normal architecture with no tachyzoites seen within the hepatocytes [56].

**Figure 10:**
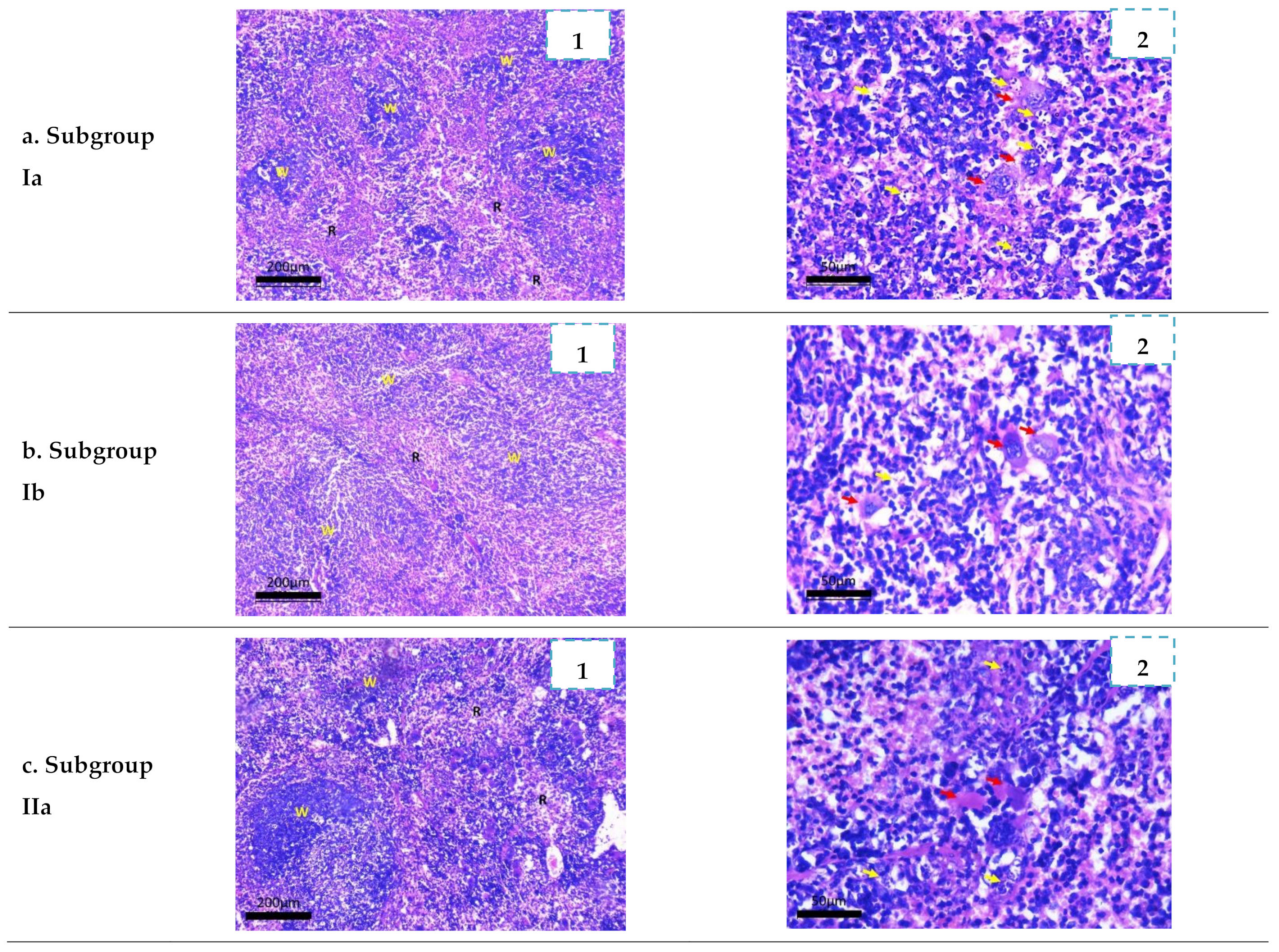

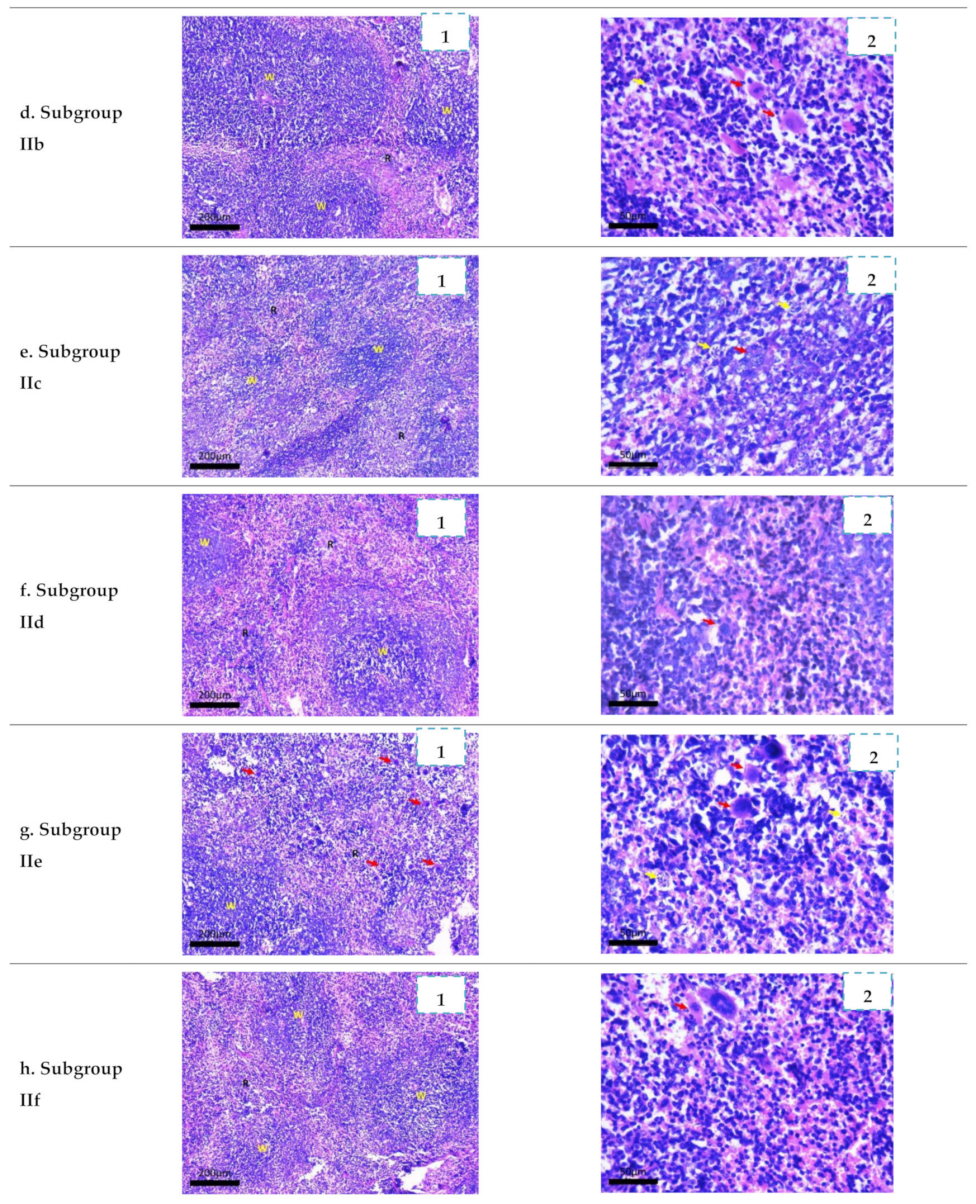
Histopathological spleen sections of different *Toxoplasma*-infected subgroups: (**a**) Subgroup Ia: (**a1**) Regression of white pulp (W) and expanded congested red pulp (R) (100×). (**a2**) Red pulp showing microgranulomas composed of scattered epithelioid histiocytes (red arrows) loaded with intracytoplasmic tachyzoites (yellow arrows) (400×). (**b**) Subgroup Ib: (**b1**) Residual congestion of red pulp (R) with slightly atrophic white pulp (W) (100×). (**b2**) Few histiocytes (red arrows) with focally detected tachyzoites (yellow arrow) (400×). (**c**) Subgroup IIa: (**c1**) Minimal improvement. Red pulp (R) is still congested on expanse of white pulp (W) (100×). (**c2**) Numerous epithelioid cells (red arrows) with evident tachyzoites (yellow arrows) (400×). (**d**) Subgroup IIb: (**d1**) Expansion of white pulp (W) and less red pulp congestion (R) (100×). (**d2**) Evident histiocytes (red arrows) with rarely detected tachyzoites (yellow arrow) (400×). (**e**) Subgroup IIc: (**e1**) Expansion of white pulp W) and less red pulp congestion (R) (100×). (**e2**) Few histiocytes (red arrows) with rarely detected tachyzoites (yellow arrows) (400×). (**f**) Subgroup IId: (**f1**) Restoration of normal splenic architecture with normal white pulp (W) and red pulp (R) (100×). (**f2**) Rare histiocytes (red arrows) with no detected tachyzoites (400×). (**g**) Subgroup IIe: (**g1**) Improvement of white pulp (W) and less congestion of red pulp (R) (100×). (**g2**) Rare histiocytes (red arrows) with few tachyzoites (yellow arrows) (400×). (**h**) Subgroup IIf: (**h1**) Restoration of normal splenic architecture with normal white pulp (W) and red pulp (R) (100×). (**h2**) Few histiocytes (red arrows) with no detected tachyzoites (400×).

**b. Spleen:** Histopathological examination of spleen sections of the infected untreated control (subgroup Ia) revealed disorganized architecture. The white pulp showed hyperplastic lymphoid follicles. They were of variable sizes with activated germinal centers in most of them. Red pulp was widened with evident congestion and increased megakaryocytic count. Sinusoids were lined by hyperplastic littoral cells. Numerous non necrotizing granulomas were seen formed of large epithelioid histiocytes loaded with tachyzoites. Capsule was thickened and infiltrated by lymphocytes (Figure 10a1,a2).

Minimal improvement was seen in subgroup IIa (Figure 10c1,c2). Red bulb congestion and granulomas are still seen. Its effect was improved when the drug was loaded on chitosan nanoparticles (subgroup IId) (Figure 10f1,f2). Moderate improvement was seen among subgroup IIb and subgroup IIc (Figure 10d1,d2,e1,e2) and their effect was maximum when loaded on chitosan nanoparticles (subgroup IIe and subgroup IIf) (Figure 10g1,g2,h1,h2). The effect of the treated subgroup subgroup IIf was comparable to the commercially used sulfadiazine (Figure 10b1,b2). This result was in agreement with El Temsahy et al. [52] who reported hyperplasia in lymphoid follicles with dilated congested sinusoids and multinucleated giant cells indicating a severe degree of inflammatory changes secondary to infection with RH strain [52]. Lee et al. [57] reported that the spleen is a very important organ that can be infected by tachyzoites of *T. gondii*, and parasitic infiltration and apoptosis induction can occur even with a very low infection dose. This can explain severe distortion in splenic architecture by infection [57]. Allam et al. [56] reported persistence of extramedullary hematopoiesis in the spleen of infected mice with RH strain. At the same time, the groups treated with spiramycin-CS NPs either in 400 mg/kg or 100 mg/kg revealed complete disappearance of necrosis and restoration of close to normal architecture. There was regeneration of white pulp, well-structured lymphoid follicles with reactive germinal centers and healthy demarcated red pulp [56].

**Figure 11:**
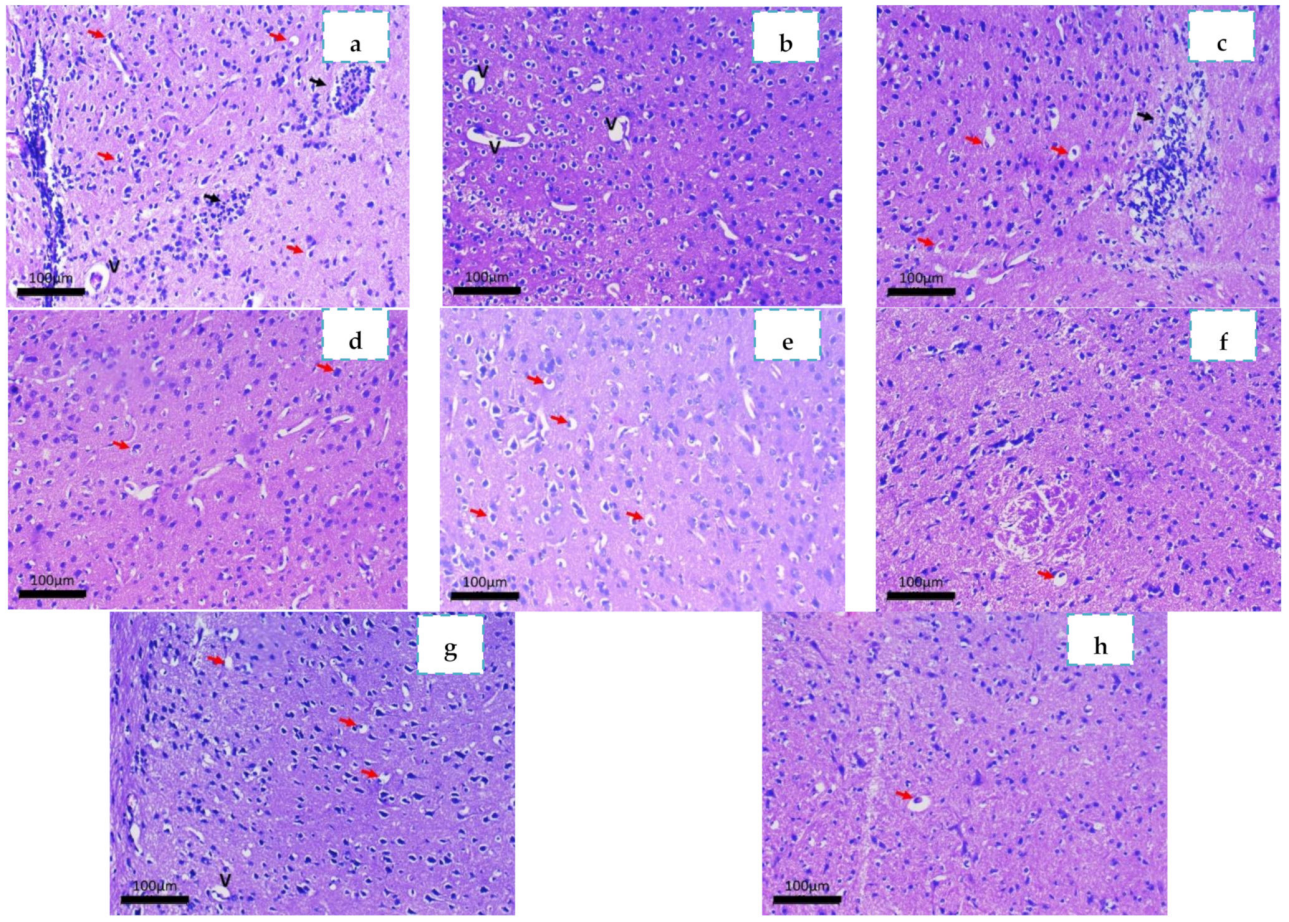
Brain section of different Toxoplasma-infected models: (**a**) Subgroup Ia: Reveals multiple foci of neuronal degenerative change (red arrows) and gliotic nodules (black arrow). Peri vascular edema was seen (V). (**b**) Subgroup Ib: Marked improvement was noted. Only few degenerated neurons were seen (red arrows) but perivascular edema is still seen (V). (**c**) Subgroup IIa: No improvement was detected. Multiple foci of neuronal degenerative change were still noted (red arrows) and gliotic nodule (black arrow). (**d**) Subgroup IIb: Marked improvement was noted. Only few degenerated neurons were seen (red arrows). (**e**) Subgroup IIc: Moderate improvement was noted. Some degenerated neurons were seen (red arrows). (**f**) Subgroup IId: Marked improvement was noted. Only few degenerated neurons were seen (red arrows). (**g**) Subgroup IIe: Moderate improvement was noted. Some degenerated neurons were seen (red arrows). Focal perivascular edema is seen (V). (**h**) Subgroup IIf: Marked improvement was noted. Only few degenerated neurons were seen (red arrows) (H&E, 200x).

**c. Brain:** Examination of subgroup Ia showed evident perivascular inflammatory cuffing composed of mononuclear cells. Perivascular edema was also noted. Gliotic nodules were seen as well as neuronal degenerative changes. Toxoplasmosis pseudocysts were not detected by light microscopy. Mononuclear cuffing’s were not detected by light microscopy in the studied cases. (Figure 11a). This was in agreement with a result reported by El Temsahy et al. [52] where heavy inflammatory infiltrates were noticed in the cortex of infected mice secondary to infection with RH strain [52]. With regard to the sulfadiazine-treated subgroup Ib, marked improvement was noted. Only few degenerated neurons were seen but perivascular edema was still observed (Figure 11b). The subgroup treated with 3a analogue showed no improvement of histologic changes as gliotic nodules and neuronal degenerative changes were still noticed (Figure 11c). Both 3b and 3c treated subgroup analogueanalogue-treated subgroups showed moderate improvement (Figure 11d,e) while the nanoparticles formulation improved the effect of all the tested drugs, especially the subgroup treated with 3c sulfadiazine (Figure 11f–h). No gliosis was detected and neuronal degenerative changes were only rarely detected. The effect of all loaded drugs on chitosan nanoparticles was similar to the commercially used sulfa. In agreement with this result was that reported by Allam et al. [56] who noted an enhancement amelioration of the encephalitis induced by the *Toxoplasma* infection after treatment with spiramycin-loaded chitosan nanoparticles.

## 3. Materials and Methods

### 3.1. Chemistry

#### Synthesis and Characterization of Sulfonamide-Based 1,2,3-Triazoles **3a**–**c**

With stirring, a solution of copper sulfate (0.10 g) and sodium ascorbate (0.15 g) in water (10 mL) was added to a solution of propargyl alcohol (**1**) (1 mmol) in DMSO (10 mL). The appropriate sulfa azide **2a–c** (1 mmol) was then added to the reaction mixture, which was stirred at room temperature for 6–10 h. TLC (hexane-ethyl acetate) was used to monitor the reaction, and after it was completed, iced water was added to the mixture. The resulting precipitate was collected by filtration, washed with saturated ammonium chloride solution, and recrystallized from ethanol/DMF to yield the desired 1,2,3-triazoles **3a–c**.

***Characterization of 4-(4-(hydroxymethyl)-1H-1,2,3-triazol-1-yl)-N-(pyrimidin-2-yl)benzenesulfonamide (3a).*** It was obtained as white crystal in 88% yield; Mp: 185–186 °C. IR (KBr) ύ_max_/cm^−1^: 1582 (C=C), 1639 (C=N), 2899, 2947 (Al.C-H), 3b6 (Ar.C-H), 33c(NH), 3487 cm^−1^ (OH). ^1^H NMR (DMSO-*d*_6_, 400 MHz): δ_H_ = 12.10 (1H, s, N**H**SO_2_), 8.79 (1H, s, C**H**-1,2,3-triazole), 8.53 (2H, bs, Ar-**H**), 8.15 (4H, bs, Ph-**H**), 7.07 (1H, bs, Ar-**H**), 5.41 (1H, s, O**H**), 4.61 (2H, s, C**H_2_**NH_2_). ^13^C NMR (DMSO-*d*_6_, 100 MHz): δ_C_ = 158.95, 158.85, 149.93, 144.56, 138.75, 127.99, 127.78, 121.65, 121.58, 120.42, 120.27 (**C**=N, Ar-**C**), 55.16 (**C**H_2_). Calculated for C_13_H_12_N_6_O_3_S: C: 46.98; H: 3.64; N: 25.29. Found: C: 46.69; H: 3.35; N: 25.08. HRMS (ESI): 332.0443 [M^+^].

***Characterization of 4-(4-(hydroxymethyl)-1H-1,2,3-triazol-1-yl)-N-(pyridin-2-yl)benzenesulfonamide (3b).*** It was obtained as yellow crystal in 86% yield; Mp: 223–224 °C. IR (KBr) ύ_max_/cm^−1^: 1577 (C=C), 1642 (C=N), 2889, 2956 (Al.C-H), 3082 (Ar.C-H), 3323(NH), 3498 cm^−1^ (OH). ^1^H NMR (DMSO-*d*_6_, 400 MHz): δ_H_ = 12.58 (1H, s, N**H**SO_2_), 8.80 (1H, s, C**H**-1,2,3-triazole), 8.13 (4H, bs, Ph-**H**), 6.87–7.20 (4H, m, Ar-**H**), 5.38 (1H, s, O**H**), 4.62 (2H, s, C**H_2_**NH_2_). ^13^C NMR (DMSO-*d*_6_, 100 MHz): δ_C_ = 169.90, 163.73, 158.90, 157.86, 149.82, 147.99, 139.26, 132.93, 129.40, 128.47, 121.21, 120.12, 119.99 (**C**=N, Ar-**C**), 55.31 (**C**H_2_). Calculated for C_14_H_13_N_5_O_3_S: C: 50.75; H: 3.95; N: 21.14. Found: C: 50.38; H: 3.55; N: 21.47. HRMS (ESI): 331.0598 [M^+^].

***Characterization of N-(diaminomethylene)-4-(4-(hydroxymethyl)-1H-1,2,3-triazol-1-yl)benzenesulfonamide (3c).*** It was obtained as yellow pale crystal in 90% yield; Mp: 254–256 °C. IR (KBr) ύ_max_/cm^−1^: 1577 (C=C), 1642 (C=N), 2889, 2956 (Al.C-H), 3082 (Ar.C-H), 3319–3376 (NH_2_), 3509 cm^−1^ (OH). ^1^H NMR (DMSO-*d*_6_, 400 MHz):δ_H_ = 8.78 (1H, s, C**H**-1,2,3-triazole), 8.05 (2H, d, *J* = 4Hz, Ar-**H**), 7.93 (2H, d, *J* = 4 Hz, Ar-H), 6.90 (4H, s, 2xN**H_2_**), 5.45 (1H, s, O**H**), 4.62 (2H, s, C**H_2_**). ^13^C NMR (DMSO-*d*_6_, 100 MHz):δ_C_ = 158.31, 149.42, 144.22, 144.02, 138.58, 127.75, 121.87, 120.59, 119.99 (**C**=N, Ar-**C**), 55.54 (**C**H_2_). Calculated for C_10_H_12_N_6_O_3_S: C: 40.54; H: 4.08; N: 28.36. Found: C: 40.89; H: 4.37; N: 28.59. HRMS (ESI): 296.0338 [M^+^].

### 3.2. Bioassay

#### 3.2.1. Nanoparticles Preparation

0.5 g of chitosan (100–150 kDa, DDa ≈ 85%, Sigma-Aldrich, Saint Louis, MO, USA) was dissolved in 100 mL of acetic acid solution (2% *v*/*v*) then stirred for 30 min and filtered using Whatman filter paper no. 1. Sodium tri-poly phosphate (TPP) solution (0.2% *w*/*v*) was prepared using deionized water then the **3a**, **3b** and **3c** solutions (40 mg) were added to the prepared TPP solution individually. Each mixture was then poured drop-wisely to the prepared chitosan solution followed by continuous stirring for 30 min followed by ultracentrifugation (25,000 rpm for 20 min). The resultant precipitate was stored at 4 °C in sterile falcon tubes for further investigations. All the chemicals were purchased from Sigma-Aldrich (Saint Louis, MO, USA).

#### 3.2.2. Characterization of the Nanoformulations

Dynamic light scattering technique was used to determine the particle size (PS), polydispersity index (PDI) and ζ potential of the prepared nanoformulae by using Malvern Zetasizer. Transmission electron microscopic (TEM) examination was used to detect the ultra-structure, size and shape of the prepared nanoformulae [58]. Each nanoformulae were lyophilized and weighed before further analyses.

#### 3.2.3. Determination of the Loading and Entrapment Efficiencies

The loading and entrapment efficiencies of the prepared nanoformulae were assessed by diluting each nanoformulae with phosphate-buffered solution (PBS) in a ration 1 to 10 v/v and then the diluted samples were centrifuged for 15 min at 15,000 rpm. UV/Vis spectroscopy (Spekol 1300, Analytik Jena, Jena, Germany) was used to measure the percentage of the loaded and unentrapped drug in each supernatant separately with methanol as a blank (at 280 nm) [58]. The percentage of the entrapment efficiency (*EE*%) was calculated according to Equation (1). While the percentage of the loading efficiency (*LE*%) was calculated according to Equation (2)

(1) EE%=Total initial drug−Total unentraped drug/Total initial drug×100

(2) LE%=Total entraped drug/Nanoparticles weight×100

### 3.3. Experminal Design and Animal Grouping

#### 3.3.1. Parasite

*T. gondii* RH virulent strain was used in the present study. The strain was obtained from the Parasitology Department, Faculty of Medicine, Alexandria University, Alexandria, Egypt. The tachyzoites were maintained through their serial intraperitoneal passage into Swiss albino mice. On the 4th day post-infection, the mice were sacrificed and their peritoneal fluid was washed three times with saline. Using a hemocytometer, the tachyzoites number in the peritoneal exudate was adjusted to be utilized for infecting mice at a dose of 2500 tachyzoites/100 μL saline /mouse.

#### 3.3.2. Drugs

In this study, the three newly synthetized sulfonamide-1,2,3-triazole hybrids **3a–c** were tested. Furthermore, the tested compounds were incorporated in chitosan nanoparticles and their effects were also assessed. The effect of the blank chitosan nanoparticles on toxoplasmosis was previously assessed showing no absolute parasite reduction but improved the efficacy of the loaded drug. All drugs were administered orally using the oral gavage technique from day zero of infection for seven successive days. The dose per mouse was dissolved in 100 μL saline for oral administration [2,59].

#### 3.3.3. Grouping of Animals and Experimental Design

Eighty laboratory-bred male Swiss Albino mice were purchased from the animal house of Pharos University in Alexandria, Egypt. Each mouse was 6–8 weeks old and 20–25 g at the start of the experiment. This animal study was approved by the Ethics Committee of Alexandria University (0104837). Each mouse was infected with the RH strain intraperitoneally in a dose of 2500 tachyzoites/100 μL.

Mice were divided into two main groups as follows:

- Group **I**: Control group (20 mice):

It was subdivided into two subgroups:

- Subgroup **Ia** (10 mice): RH-infected untreated control [51].

Each mouse received 100 μL oral saline (the vehicle of the used drugs) by gavage needle starting from the day zero of infection for seven successive days.

- Subgroup **Ib** (10 mice): RH-infected mice treated with sulfadiazine in a dose of 320 mg/kg/day for seven successive days [52].

- Group **II**: Experimental group (60 mice):

It was subdivided into six treated subgroups (10 mice each). All synthetized sulfadiazine analogues were suspended in 100 μL saline/dose/mouse and were orally administered by gavage needle from the start of infection for seven days in a dose of 320 mg/kg/day.

- Subgroup **IIa**: RH-infected mice treated with **3a** sulfadiazine analogue.

- Subgroup **IIb**: RH-infected mice treated with **3b** sulfadiazine analogue.

- Subgroup **IIc**: RH-infected mice treated with **3c** sulfadiazine analogue.

- Subgroup **IId**: RH-infected mice treated with nano-loaded **3a** sulfadiazine analogue.

- Subgroup **IIe**: RH-infected mice treated with nano-loaded **3b** sulfadiazine analogue.

- Subgroup **IIf**: RH-infected mice treated with nano-loaded **3c** sulfadiazine analogue.

- Five mice of each subgroup (either control or experimental) were observed daily for early detection of abnormal feeding behavior. Survival time was recorded for thirty days post-infection.
- The other five mice were anesthetized and sacrificed by cervical dislocation on the 8th day post-infection (24 h after the last dose of treatment). Liver, spleen and brain were obtained from each mouse for further parasitological assessment. Peritoneal exudate from each mouse was fixed in glutaraldehyde for further morphological studies using scanning electron microscope. Specimens from liver, spleen and brain tissues were fixed in formalin for histopathological studies.

### 3.4. Evaluation of Drug Efficacy as Anti-Parasitic Agent

Assessment of each drug efficacy was carried out by clinical, parasitological and morphological studies as follows:

#### 3.4.1. Clinical Study

Mice were observed daily to identify any changes in the clinical behavior (attitude and posture) and food intake [2,54].

#### 3.4.2. Parasitological Study

##### Survival Rate

Five mice, from each studied subgroup, were observed daily to determine the percentage of mice living over time through Kaplan–Meier survival curve [2,60].

##### Parasite Load

Giemsa-stained impression smears of the liver, spleen and brain were prepared for counting the tachyzoites. The mean count of tachyzoites of twenty different oil immersion fields from each organ of each mouse was then obtained (ten fields/each slide and two slides/each organ). The tachyzoites mean number in each subgroup of mice was estimated [2,55,61].

##### Percent Reduction (%R) in Parasite Load

The % R in the parasite load (mean tachyzoites count) in the liver, spleen and brain was calculated according to the following equation [54]:

$$\%R=\frac{\mathrm{Mean}\;\mathrm{count}\;\mathrm{in}\;\mathrm{infected}\;\mathrm{untreated}\;\mathrm{control}\;group-\mathrm{Mean}\;\mathrm{count}\;\mathrm{in}\;experimental\;group}{\mathrm{Mean}\;\mathrm{count}\;\mathrm{in}\;\mathrm{infected}\;\mathrm{untreated}\;\mathrm{control}\;\mathrm{group}}\times \;100$$

#### 3.4.3. Morphological Study by Light Microscopy and Scanning Electron Microscopy (SEM)

The peritoneal fluid of all subgroups was collected on the sacrifice day and examined by the light microscopy at 400× to demonstrate the effect of treatment on the movement of *T. gondii* tachyzoites. The peritoneal fluid was then fixed in glutaraldehyde and prepared for examining the ultra-structure of the parasites by SEM (JSM-IT 200, JOEL, Tokyo, Japan) [62].

#### 3.4.4. Histopathological Study

The liver, spleen, and brain of each studied group were collected after the mice were sacrificed. Different tissues were fixed in 10% formalin and processed into paraffin blocks. Four microns sections were cut and mounted on glass slides for histopathologic assessment. Hematoxylin and eosin (H&E) stain was used. Liver sections were assessed for architecture, degree of portal inflammation (mild, moderate, and marked) and scored according to the numbers of inflammatory foci (focal lytic necrosis) in ten 400xpower fields while searching for *Toxoplasma* pseudocyst using light microscope [63]. Spleen sections were assessed for architecture, changes in red and white pulp and presence/absence of tachyzoites. Brain sections were also examined in different groups [64,65].

### 3.5. Statistical Analysis of the Data

Data were fed to the computer and analyzed using IBM SPSS software package version 20.0. (IBM Corp, Armonk, NY, USA). The Kolmogorov–Smirnov was used to verify the normality of distribution of variables. ANOVA was used for comparing the studied groups and followed by the post hoc test (Tukey) for pairwise comparison. Kaplan–Meier survival curve was used for overall survival. Significance of the obtained results was judged at the 5% level [66,67].

## 4. Conclusions

Novel sulfoamide-1,2,3-triazole molecular hybrids were successfully designed and synthesized using Cu(I)-catalyzed 1,3-dipolar cycloaddition between propargyl amine and appropriate sulfa drug azides. After full spectroscopic characterization, the newly synthesized 1,2,3-triazole-sulfa molecular conjugates were investigated for their Toxoplasma efficiency. All synthesized sulfa derivatives **3a–c** revealed a marked effectiveness in the treatment of the virulent toxoplasma RH strain. The observed difference in the biological activity between the sulfonamide analogues could be attributed to the difference in the chemical composition where **3a**, **3b** and **3c** had azine, diazine and guanidine linked to substituted 1,2,3-triazole ring, respectively. Moreover, the study illustrated state-of-the-art chitosan NPs used to convey the newer sulfonamide-1,2,3-triazole derivatives successfully to different tissues and bypass the blood–brain barrier. Remarkably, the synthesized sulfonamide-1,2,3-triazole **3c** nanoformula can be further considered as a potential candidate for toxoplasmosis treatment as it achieved 100% mice survival, 100% parasite reduction as well as exhibiting a marked histopathological improvement in the examined organs.

## Figures and Tables

**Table 1:** Particle size (PS), zeta potential (ζ potential), percentage of the entrapment efficiency (EE%), percentage of loading efficiency (LE%) and polydispersity index (PDI) of the prepared nanoformulae.

Nano Formulations	PS (nm)	ζ Potential (mV)	PDI	Entrapment Efficiency % (EE%)	Loading Efficiency (LE%)
NCs-3a	76.3 ± 10.9	+36.4 ± 2.7	0.34 ± 0.1	78.11 ± 1.70	38.42 ± 0.8
NCs-3b	50.4 ± 7.3	+37.2 ± 0.8	0.36 ± 0.03	80.15 ± 1.10	38.70 ± 0.5
NCs-3c	36.0 ± 10.4	+39.3 ± 1.9	0.35 ± 0.1	89.27 ± 1.23	43.31 ± 1.0

**Table 2:** Kaplan-Meier survival for all subgroups.

Subgroup	Mean Survival	% End of Study	Log Rank	
			χ^2^	*p*
**Ia**	6.6	0.0	81.343 *	<0.001 *
**Ib**	29.0	80.0		
**IIa**	11.8	0.0		
**IIb**	12.2	0.0		
**IIc**	13.0	0.0		
**IId**	16.8	0.0		
**IIe**	27.4	60.0		
**IIf**	30.0	100.0		

*: Statistically significant at *p* ≤ 0.05.

**Table 3:** Log rank survival pairwise comparison between the different studied subgroups.

Subgroup	Ib	IIa	IIb	IIc	IId	IIe	IIf
**Ia**	0.003 *	0.003 *	0.003 *	0.003 *	0.003 *	0.003 *	0.003 *
**Ib**		0.002 *	0.003 *	0.003 *	0.003 *	0.459	0.317
**IIa**			0.549	0.049 *	0.002 *	0.002 *	0.002 *
**IIb**				0.134	0.003 *	0.003 *	0.003 *
**IIc**					0.003 *	0.003 *	0.003 *
**IId**						0.003 *	0.003 *
**IIe**							0.134

*: Statistically significant at *p* ≤ 0.05.

**Table 4:** Mean tachyzoites count and percent reduction in the liver, spleen and brain of the different studied subgroups.

	Subgroup Ia (*n* = 5)	Subgroup Ib (*n* = 5)	Subgroup IIa (*n* = 5)	Subgroup IIb (*n* = 5)	Subgroup IIc (*n* = 5)	Subgroup IId (*n* = 5)	Subgroup IIe (*n* = 5)	Subgroup IIf (*n* = 5)
**Liver**								
**Mean**	10.74	0.04 ^#^	1.96 ^#^	1.72 ^#^	1.17 ^#^	1.07 ^#^	0.66 ^#^	0.0 ^#^
**±SD.**	1.84	0.07	0.80	0.54	0.69	1.84	0.81	0.0
**F (*p*)**	56.471 * (<0.001 *)							
**% Reduction**		99.63	81.75	83.99	89.11	90.04	93.85	100.0
**Spleen**								
**Mean**	14.74	0.04 ^#^	2.32 ^#^	2.01 ^#^	1.56 ^#^	1.45 ^#^	0.04 ^#^	0.0 ^#^
**±SD.**	2.61	0.09	0.49	2.38	0.70	3.16	0.09	0.0
**F (*p*)**	41.833 * (<0.001 *)							
**% Reduction**		99.73	84.26	86.36	89.42	90.16	99.73	100.0
**Brain**								
**Mean**	4.52	0.0 ^#^	0.80 ^#^	0.81 ^#^	0.61 ^#^	0.26 ^#^	0.12 ^#^	0.0 ^#^
**±SD.**	1.08	0.0	0.67	0.83	0.65	0.44	0.22	0.0
**F (*p*)**	30.448 * (<0.001 *)							
**% Reduction**		100.0	82.30	82.08	86.50	94.25	97.35	100.0

Data were expressed using Mean ± SD. F: F for ANOVA test. Pairwise comparison between each 2 groups was carried out using the post hoc test (Tukey). *p*: *p* value for comparing between the studied groups. *: Statistically significant at *p* ≤ 0.05. ^#^: Significant with subgroup **Ia**.

## Data Availability

No data was reported.
